# Three-dimensional rotational angiography–based multimodal fusion imaging for preoperative neurovascular assessment in intracranial meningiomas: a retrospective technical feasibility study of 72 cases

**DOI:** 10.3389/fneur.2026.1873767

**Published:** 2026-08-26

**Authors:** Yue Ma, Zhouyang Zhao, Yimei Yang, Xiaobin Zhang, Shifeng Deng, Lijin Huang

**Affiliations:** Department of Neurosurgery, The Second Clinical College of Guangzhou University of Chinese Medicine, The Second Affiliated Hospital of Guangzhou University of Chinese Medicine, Guangdong Provincial Hospital of Chinese Medicine, Guangzhou, Guangdong, China

**Keywords:** 3D rotational angiography, intracranial meningioma, multimodal fusion imaging, neurovascular mapping, skull base, surgical planning

## Abstract

**Background and objective:**

Surgical management of anatomically complex intracranial meningiomas requires accurate assessment of tumor vascular supply, venous sinus involvement, cortical venous relationships, and skull base anatomy. Although multimodal fusion imaging and angiographic assessment are established, the incremental role and appropriate use of arterial- and venous-phase three-dimensional rotational angiography (3DRA)-based multimodal fusion imaging (MFI) remain uncertain. We evaluated the technical feasibility, anatomical information yield, and potential decision-support utility of this workflow.

**Methods:**

We retrospectively reviewed 72 consecutive patients with pathologically confirmed intracranial meningiomas who underwent microsurgical resection with preoperative 3DRA-based MFI between May 2023 and March 2026. Preoperative imaging datasets included contrast-enhanced three-dimensional T1-weighted magnetic resonance imaging (MRI), thin-section computed tomography (CT), and arterial- and venous-phase 3DRA. These datasets were fused to generate patient-specific three-dimensional models for surgical planning, neurovascular mapping, early intraoperative anatomical orientation, and postoperative assessment. Simpson grade I-II resection, perioperative complications, and digital subtraction angiography (DSA)-related complications were recorded.

**Results:**

Among the 72 patients, 35 tumors were located at the skull base and 37 were non-skull base meningiomas. Venous sinus involvement was identified in 24 patients, pial arterial supply in 14 patients, and meningeal arterial supply in 54 patients. Abnormal vascular findings were detected in 10 patients, including 9 aneurysms and 1 arterial occlusion. Simpson grade I-II resection was achieved in 67 patients (93.1%), and perioperative complications occurred in 5 patients (6.9%). No DSA-related complications were observed. In exploratory subgroup analyses, no statistically significant differences in resection or complication rates were observed according to angiographically defined vascular involvement or tumor location.

**Conclusion:**

3DRA-based MFI is a feasible adjunct for patient-specific neurovascular and skull base assessment in selected patients with anatomically complex intracranial meningiomas. Its potential value may be greatest in skull base, hypervascular, vessel-adjacent, and venous sinus-related tumors when detailed vascular information may influence surgical or embolization planning. Because this retrospective study lacked a control group and did not prospectively assess management changes, operative efficiency, functional outcomes, or cost-effectiveness, it does not establish superiority over noninvasive imaging or support routine use in all meningioma patients. Future prospective comparative studies should include an appropriate conventional-imaging control group, prospectively document management plans before and after review of the fusion images, and assess predefined operative, functional, safety, and cost-effectiveness outcomes to determine the incremental clinical value of this approach.

## Introduction

Intracranial meningiomas are among the most common primary tumors of the central nervous system and are primarily treated surgically when symptomatic, enlarging, or anatomically complex. Successful treatment requires maximal safe tumor removal together with careful management of the dural attachment, skull base anatomy, arterial supply, cortical veins, venous sinuses, and adjacent neurovascular structures. These relationships are particularly important in skull base, parasagittal, falcine, tentorial, torcular, and vessel-adjacent meningiomas, where the extent of resection must be balanced against the risk of vascular or neurological injury (1–4). MRI provides essential information regarding tumor extent, brain edema, and adjacent neural structures, whereas CT is useful for assessing calcification, skull base anatomy, and bone involvement. CTA and MRA provide noninvasive vascular assessment, while DSA remains valuable for evaluating tumor-feeding arteries, venous sinus patency, cortical venous drainage, collateral pathways, and associated vascular abnormalities.

Multimodal image fusion enables simultaneous visualization of tumor morphology, bone structures, arteries, veins, venous sinuses, and surrounding anatomical landmarks (5–7). However, conventional MRI, CT, CTA, MRA, and projection-based DSA each provide only part of the information required for complex surgical planning. In selected cases, integration of arterial- and venous-phase 3DRA with MRI and CT may improve the spatial representation of small feeding arteries, pial supply, cortical veins, venous sinus patency, collateral venous drainage, and skull base corridors. The incremental value of this approach, the patients most likely to benefit, and its balance of benefit, invasiveness, radiation, contrast exposure, and cost require careful definition.

**Figure 1:**
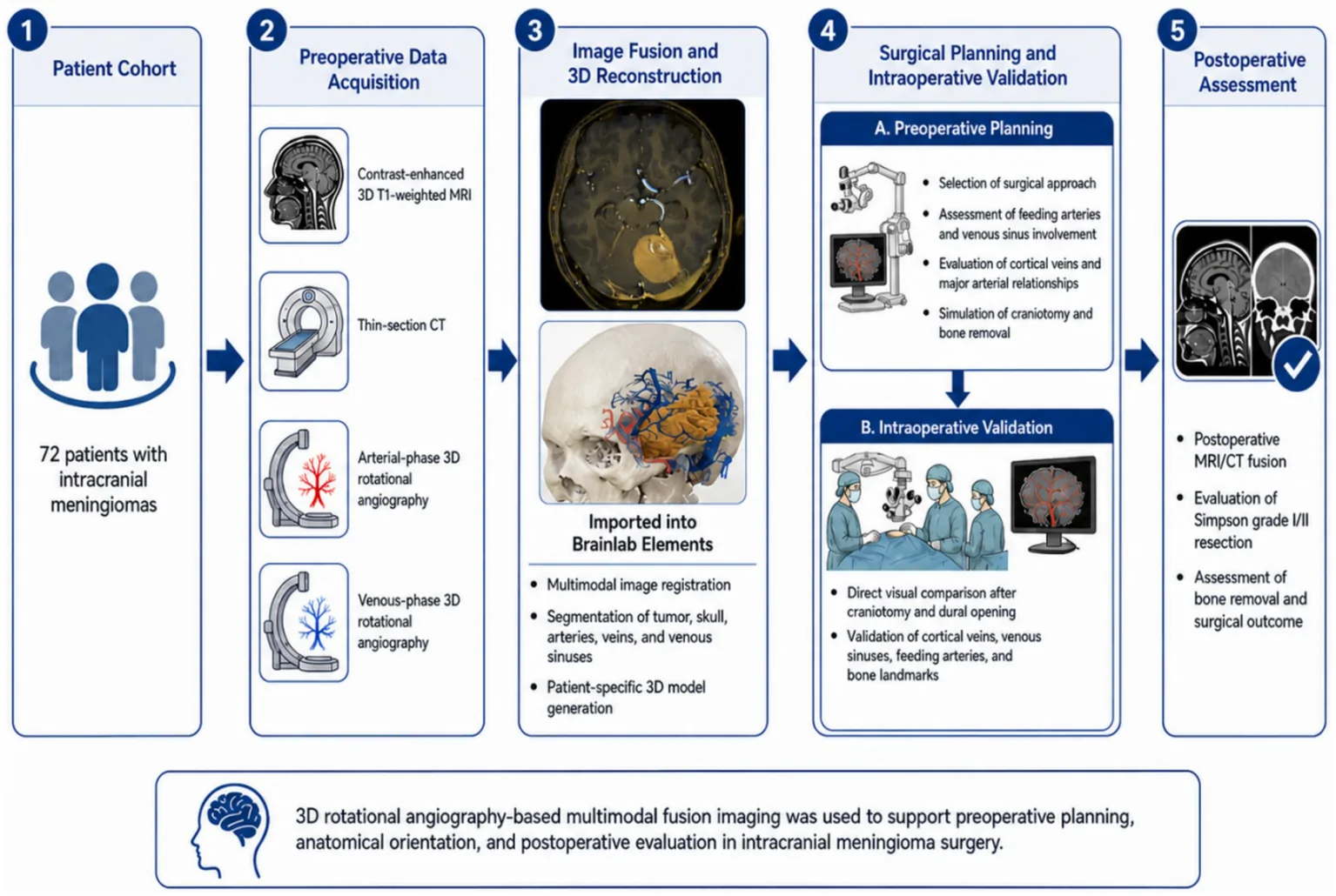
Workflow of 3DRA-based MFI in intracranial meningioma surgery. Preoperative imaging datasets, including contrast-enhanced 3D T1-weighted MRI, thin-section CT, arterial-phase 3DRA, and venous-phase 3DRA, were imported into brainlab elements for image fusion and 3D reconstruction. Patient-specific MFI models were used for preoperative surgical planning, intraoperative visual validation after craniotomy and dural opening, and postoperative assessment.

To address these issues, we developed a standardized workflow (Figure 1) integrating contrast-enhanced MRI, thin-section CT, arterial-phase 3DRA, and venous-phase 3DRA into patient-specific three-dimensional models. This retrospective study evaluated the technical feasibility, anatomical information provided, and potential planning utility of the workflow in 72 consecutive patients who underwent 3DRA-based MFI. The study was not designed to demonstrate superiority over conventional imaging or improvement in clinical outcomes.

## Methods

### Study design

This was a retrospective single-center technical feasibility study evaluating the integration of arterial- and venous-phase 3DRA with MRI and CT for patient-specific neurovascular mapping and anatomical decision support in intracranial meningioma surgery.

### Patient cohort

Between May 2023 and March 2026, 72 consecutive patients with intracranial meningiomas who underwent microsurgical resection with preoperative 3DRA-based MFI at our institution were retrospectively reviewed.

All included patients had pathologically confirmed intracranial meningiomas and complete preoperative imaging datasets, including contrast-enhanced three-dimensional T1-weighted magnetic resonance imaging (MRI), thin-section computed tomography (CT), and arterial- and venous-phase digital subtraction angiography (DSA)-based 3DRA.

Patients were excluded if they had incomplete imaging data, unavailable postoperative imaging, or non-meningioma pathology. The decision to perform DSA and 3DRA-based MFI was made by the treating surgical team according to clinical judgment rather than a predefined universal protocol; the examination was not intended as routine assessment for all patients with meningioma. This study was approved by the Ethics Committee (Guangdong Provincial Hospital of Chinese Medicine), and written informed consent was obtained from all enrolled patients.

### Data acquisition

Preoperative imaging data were collected using MRI, CT, and DSA. These datasets were subsequently integrated to generate patient-specific three-dimensional multimodal fusion models for surgical planning and intraoperative anatomical orientation.

#### MRI

MRI was performed using a Siemens MAGNETOM Prisma 3.0 T scanner (Erlangen, Germany). Contrast-enhanced three-dimensional T1-weighted MPRAGE images were acquired for tumor and brain anatomical visualization. The sequence parameters were as follows: repetition time, 2,300 ms; echo time, 2.5 ms; inversion time, 900 ms; flip angle, 9°; field of view, 256 mm; matrix, 256 × 256; isotropic voxel size, 1 mm^3^; bandwidth, 240 Hz/pixel; and acquisition time, approximately 5 min 21 s. Gadolinium contrast medium was administered intravenously at a dose of 0.1 mmol/kg, and postcontrast images were acquired 5–10 min after injection.

#### CT

CT was performed using a Siemens SOMATOM Definition AS scanner (Forchheim, Germany). Thin-section images were acquired with the following parameters: slice thickness and reconstruction interval, 1 mm; tube voltage, 120 kVp; tube current, 200–300 mAs; field of view, 250 mm; matrix, 512 × 512; and bone window reconstruction kernel, H70h. CT images were used to delineate skull base anatomy, bone involvement, and craniotomy-related anatomical landmarks.

#### DSA and 3DRA

DSA was performed using a Siemens Artis zee biplane angiography system (Erlangen, Germany) with an HDR as40 detector for 3DRA. The rotational acquisition covered an arc of 200° with an acquisition duration of 5 s.

Arterial- and venous-phase datasets were obtained in separate rotational acquisitions. For arterial-phase 3DRA, the X-ray delay was set at 1.5 s to optimize arterial opacification. For venous-phase 3DRA, the delay was adjusted to 8–12 s according to the individual circulation time to optimize visualization of the venous sinuses, cortical veins, and major venous drainage pathways.

For arterial-phase 3DRA, iohexol was injected at a rate of 2.5 mL/s, with a total volume of 17 mL, an X-ray delay of 1.5 s, and an injection duration of approximately 6.8 s.

For venous-phase 3DRA, iohexol was injected at the same rate and volume, with an X-ray delay of 8–12 s. The delay was adjusted to optimize visualization of venous sinuses and major venous drainage pathways. The acquisition protocol was standardized to improve delineation of feeding arteries, cortical veins, venous sinuses, and collateral venous drainage.

All angiographic acquisitions were performed by the same member of the surgical team, who also participated in image reconstruction and the craniotomy procedures. This arrangement helped maintain continuity among DSA acquisition, MFI reconstruction, preoperative interpretation, and intraoperative visual validation.

### Image fusion and three-dimensional reconstruction

Raw Digital Imaging and Communications in Medicine (DICOM) datasets from MRI, CT, arterial-phase 3DRA, and venous-phase 3DRA were imported into Brainlab Elements (Brainlab AG, Munich, Germany). Image registration and segmentation were performed using Image Fusion, Elements Segmentation, SmartBrush, and Viewer modules. Model quality control consisted of visual cross-checking of registration and segmentation against the source MRI, CT, and angiographic datasets before clinical use; no predefined quantitative registration-error threshold or inter-observer reproducibility assessment was applied.

Tumors, skull structures, arteries, veins, and venous sinuses were segmented and reconstructed into patient-specific MFI models. The models were used to evaluate tumor location, arterial supply, pial arterial supply, major arterial encasement, cortical venous involvement, venous sinus invasion and patency, collateral venous drainage, skull base anatomy, and abnormal vascular findings, including aneurysm and arterial occlusion. The overall workflow is illustrated in Figure 1.

### Surgical procedure and intraoperative visual validation

Before surgery, the surgical team reviewed the MFI models together with conventional MRI, CT, and DSA images. The MFI models were used to evaluate the surgical approach, tumor base, feeding arteries, venous sinus involvement, cortical venous relationships, major arterial relationships, and skull base anatomy.

After craniotomy and dural opening, the exposed anatomical structures were visually compared with the preoperative MFI models. Particular attention was paid to cortical veins, venous sinuses, feeding arteries, major arteries, and bone landmarks. All operations were performed by the same senior neurosurgeon with more than 25 years of operative experience.

### Outcome assessment

Simpson grades were determined based on operative records and contrast-enhanced postoperative MRI performed within 48 h after surgery. Simpson grade I-II resection was classified as favorable resection, and the proportion of patients achieving favorable resection was calculated.

Perioperative complications were recorded and included postoperative neurological deterioration, intracranial hemorrhage, cerebral infarction, and vascular injury requiring repair. DSA-related complications were recorded separately.

Angiographically defined vascular involvement was defined as the presence of at least one of the following findings on preoperative 3DRA-based MFI: pial arterial supply, major arterial encasement, cortical venous involvement, or venous sinus invasion. Tumor location was classified as skull base or non-skull base. Estimated blood loss, operative duration, transfusion requirements, standardized functional outcome scores, radiation dose, cost, and prospectively defined changes in surgical approach or embolization planning were not systematically recorded and were therefore not analyzed.

### Statistical analysis

Continuous variables are presented as mean ± standard deviation and range. Categorical variables are presented as numbers and percentages. The Simpson grade I-II resection rate and complication rate were compared between subgroups according to angiographically defined vascular involvement and tumor location. Because of the relatively small number of events in some cells, Fisher’s exact test was used. These subgroup comparisons were exploratory. A two-sided *p*-value <0.05 was considered statistically significant. Statistical analyses were performed using SPSS version 22.0 software.

## Results

### Patient characteristics

Preoperative 3DRA-based MFI was successfully completed in all 72 patients. The cohort included 53 female and 19 male patients, with a mean age of 57.8 ± 10.5 years (range, 36–83 years). The mean maximum tumor diameter was 4.05 ± 1.48 cm (range, 0.9–7.8 cm). Pathological examination confirmed WHO grade 1 meningioma in 51 patients (70.8%), WHO grade 2 meningioma in 18 patients (25.0%), and WHO grade 3 meningioma in 3 patients (4.2%). Because only three tumors were WHO grade 3, grade-specific outcome analyses were not performed.

Thirty-five tumors (48.6%) were located at the skull base, including 11 anterior skull base meningiomas (15.3%), 16 middle skull base meningiomas (22.2%), and 8 posterior skull base meningiomas (11.1%). The remaining 37 tumors (51.4%) were non-skull base meningiomas, including 13 convexity tumors (18.1%), 4 torcular Herophili tumors (5.6%), 16 parasagittal or falcine tumors (22.2%), and 4 tumors at other locations (5.6%).

### Radiological and angiographic findings

T2 flow voids were observed in 34 patients (47.2%), peritumoral brain edema on T2/FLAIR images in 31 patients (43.1%), and calcification in 28 patients (38.9%). Pial arterial supply was identified in 14 patients (19.4%), and meningeal arterial supply was identified in 54 patients (75.0%).

Venous sinus involvement was identified in 24 patients (33.3%). Among these 24 patients, Sindou grade I was observed in 14 patients (58.3%), grade II in 2 patients (8.3%), grade IV in 7 patients (29.2%), and grade VI in 1 patient (4.2%). No patients were classified as Sindou grade III or V.

Abnormal vascular findings were detected in 10 patients (13.9%), including aneurysms in 9 patients (12.5%) and arterial occlusion in 1 patient (1.4%). No DSA-related complications occurred in this cohort.

The clinical, radiological, angiographic, and surgical characteristics of the cohort are summarized in Table 1.

**Table 1:** Clinical, radiological, angiographic, and surgical characteristics of 72 patients with intracranial meningiomas.

Variable	Value
Number of patients	72
Age, mean ± SD (range), years	57.8 ± 10.5 (36–83)
Sex, female/male	53/19
Maximum tumor diameter, mean ± SD, cm	4.05 ± 1.48 (0.9–7.8)
WHO grade	
1	51 (70.8%)
2	18 (25.0%)
3	3 (4.2%)
Skull base meningiomas	35 (48.6%)
Anterior skull base	11 (15.3%)
Middle skull base	16 (22.2%)
Posterior skull base	8 (11.1%)
Non-skull base meningiomas	37 (51.4%)
Convexity	13 (18.1%)
Torcular herophili	4 (5.6%)
Parasagittal/Falcine	16 (22.2%)
Other	4 (5.6%)
T2 flow void present	34 (47.2%)
Peritumoral brain edema on T2/FLAIR	31 (43.1%)
Calcification	28 (38.9%)
Pial arterial supply present	14 (19.4%)
Meningeal arterial supply present	54 (75.0%)
Venous sinus involvement	24 (33.3%)
Sindou grade I	14/24 (58.3%)
Sindou grade II	2/24 (8.3%)
Sindou grade III	0/24 (0%)
Sindou grade IV	7/24 (29.2%)
Sindou grade V	0/24 (0%)
Sindou grade VI	1/24 (4.2%)
Abnormal vascular findings	10 (13.9%)
Aneurysm	9 (12.5%)
Arterial occlusion	1 (1.4%)
Simpson grade I-II resection rate	67 (93.1%)
DSA-related complications	0 (0%)

Values are presented as *n* (%) unless otherwise indicated. Percentages for Sindou grades were calculated among patients with venous sinus involvement.

### Surgical outcomes

Simpson grade I-II resection was achieved in 67 of 72 patients, yielding an overall rate of 93.1%. Perioperative complications occurred in 5 patients, corresponding to an overall complication rate of 6.9%. These outcomes are presented descriptively because the study did not include a comparison group.

Angiographically defined vascular involvement was identified in 42 patients. Patients with angiographically defined vascular involvement had a Simpson grade I-II resection rate of 95.2% (40/42), compared with 90.0% (27/30) in patients without angiographically defined vascular involvement. The difference was not statistically significant (*p* = 0.643). The complication rates were 9.5% (4/42) and 3.3% (1/30), respectively, with no statistically significant difference between the two groups (*p* = 0.393).

When stratified by tumor location, skull base meningiomas showed a Simpson grade I-II resection rate of 88.6% (31/35), whereas non-skull base meningiomas showed a rate of 97.3% (36/37). The difference was not statistically significant (*p* = 0.193). The corresponding complication rates were 8.6% (3/35) and 5.4% (2/37), respectively, with no statistically significant difference between groups (*p* = 0.670).

Perioperative complications occurred in five patients (6.9%). One patient with a parietal perirolandic meningioma developed delayed postoperative intracranial hemorrhage, which was considered likely venous in origin; the patient subsequently achieved complete neurological recovery. One patient with a straight sinus meningioma developed postoperative cerebellar hemorrhage, considered possibly related to cerebellar retraction, and had residual ataxia. One patient with a foramen magnum meningioma developed postoperative hemiplegia, presumed to be related to compromise of a brainstem perforating artery; the patient recovered to ambulatory status at the 1-year follow-up. One patient with a central-region meningioma experienced postoperative deterioration in limb strength, possibly related to compromise of pial arterial supply, and recovered by 6 months. One patient with a tuberculum sellae meningioma developed postoperative visual deterioration, considered possibly related to compromise of small perforating vessels supplying the optic nerve; the visual deficit persisted during follow-up.

The exploratory surgical outcomes according to angiographically defined vascular involvement and tumor location are summarized in Table 2.

**Table 2:** Surgical outcomes according to angiographically defined vascular involvement and tumor location.

Variable	Subgroup	*n*	Simpson grade I-II resection, *n* (%)	Complications, *n* (%)	*p*-value for resection	*p*-value for complications
Angiographically defined vascular involvement	Present	42	40 (95.2%)	4 (9.5%)	0.643	0.393
	Absent	30	27 (90.0%)	1 (3.3%)		
Tumor location	Skull base	35	31 (88.6%)	3 (8.6%)	0.193	0.670
	Non-skull base	37	36 (97.3%)	2 (5.4%)		

Values are presented as *n* (%). *p*-values were calculated using Fisher’s exact test. Simpson grade I-II resection was defined as favorable resection. Angiographically defined vascular involvement was defined as the presence of pial arterial supply, major arterial encasement, cortical venous involvement, or venous sinus invasion identified on preoperative 3DRA-based MFI. Complications included postoperative neurological deterioration, intracranial hemorrhage, cerebral infarction, and vascular injury requiring repair. Subgroup comparisons were exploratory.

### Case illustrations

**Figure 2:**
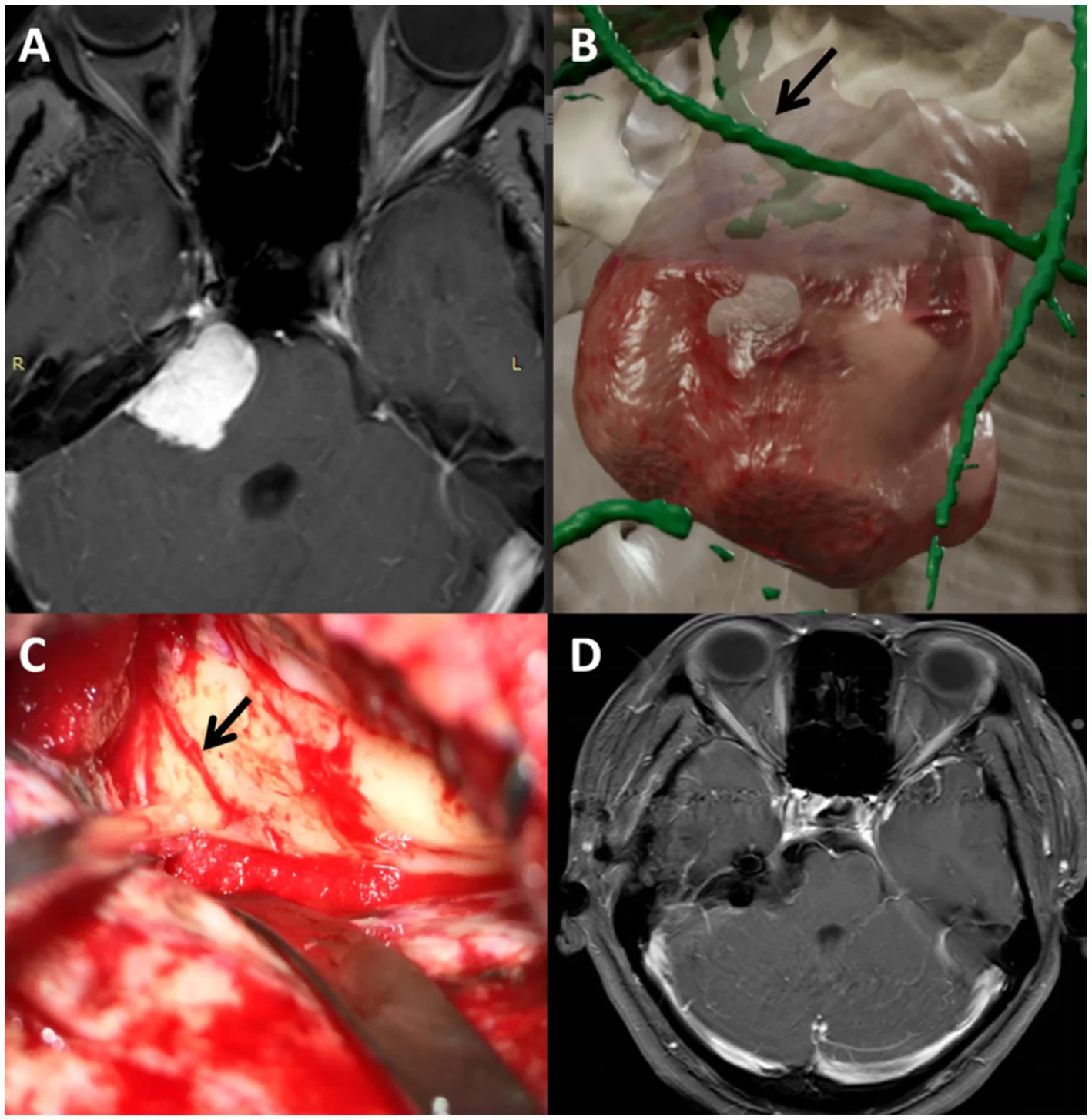
Petroclival meningioma with middle meningeal artery supply. **(A)** Preoperative contrast-enhanced MRI showing a right petroclival meningioma. **(B,C)** Preoperative MFI demonstrating tumor blood supply from the middle meningeal artery in the petrous bone region. The feeding artery is indicated by the black arrow. **(D)** Postoperative contrast-enhanced MRI showing Simpson grade I resection.

*Case 1*: A 41-year-old female presented with recurrent right facial pain for 4 months and was diagnosed with a right petroclival meningioma. Preoperative MFI demonstrated the spatial relationship between the tumor, petrous bone, and tumor-feeding arteries. The feeding artery from the middle meningeal artery was clearly visualized in the petrous bone region. These findings supported the selection of a Kawase approach and facilitated preoperative planning of petrous bone removal. Intraoperative findings confirmed the feeding artery identified on preoperative MFI, and Simpson grade I resection was achieved (Figure 2).

**Figure 3:**
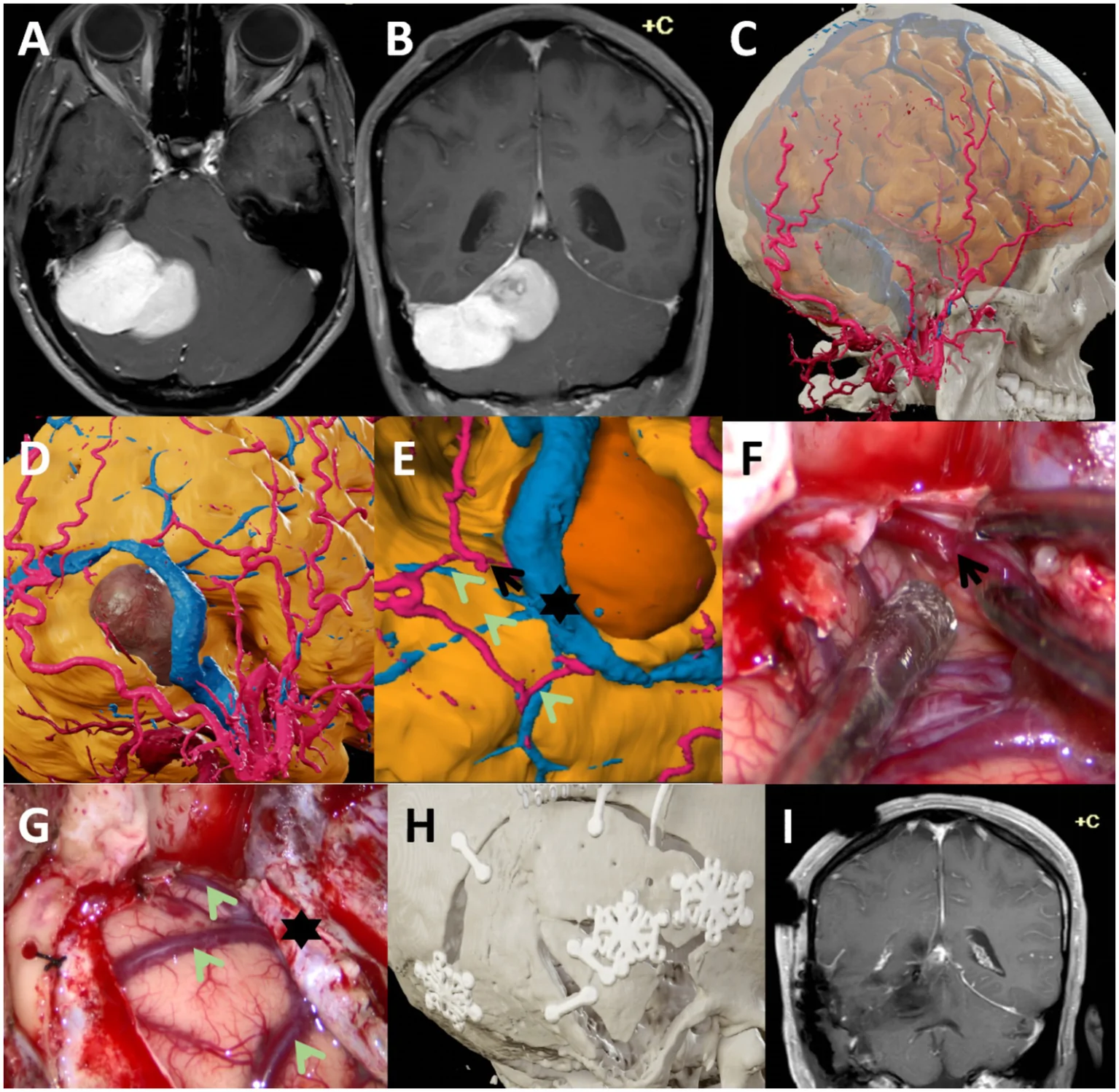
Tentorial-petrosal meningioma with complex arterial and venous relationships. **(A,B)** Preoperative MRI showing a right tentorial-petrosal meningioma. **(C–G)** MFI demonstrating arteries, veins, the sigmoid sinus, and the vein of Labbé. The vein of Labbé is indicated by the green arrow, the tumor-feeding artery from the middle meningeal artery by the black arrow, and the sigmoid sinus by the black asterisk. The preoperative reconstruction was visually consistent with intraoperative findings. **(H)** Postoperative cranial bone reconstruction. **(I)** Postoperative contrast-enhanced MRI showing Simpson grade I resection.

*Case 2*: A 38-year-old female presented with hydrocephalus and vomiting for more than 2 weeks and was diagnosed with a highly vascular right tentorial-petrosal meningioma. Preoperative MFI delineated the tumor-feeding arteries, sigmoid sinus, and vein of Labbé. The relationship between the tumor and major venous structures was visually confirmed during surgery. These findings helped guide the presigmoid approach and supported preservation of critical venous drainage pathways. Simpson grade I resection was achieved (Figure 3).

**Figure 4:**
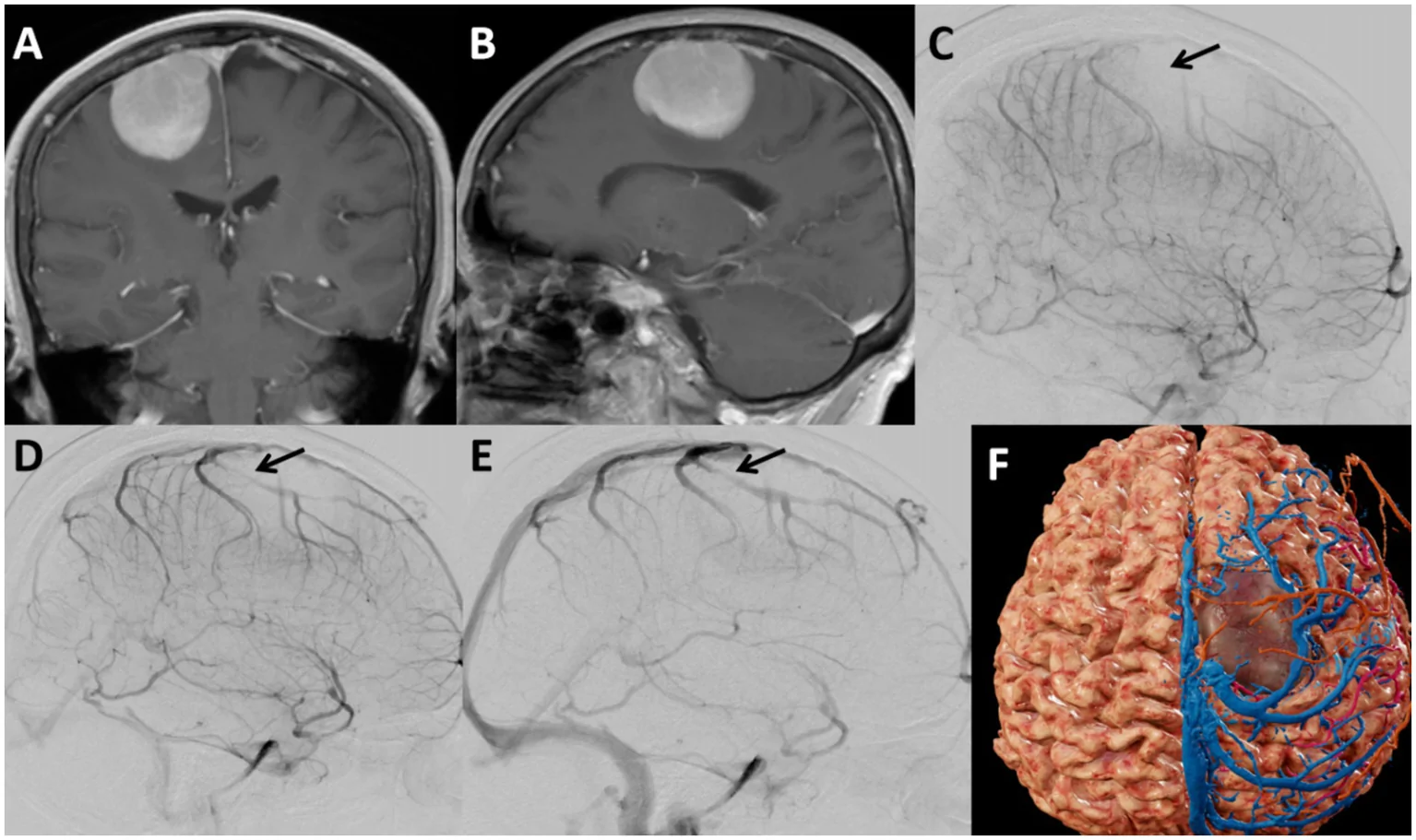
Cortical venous drainage impairment caused by meningioma-related compression. **(A,B)** Preoperative MRI showing the lesion and its relationship with adjacent cortex. **(C–E)** Venous-phase DSA demonstrating impaired cortical venous drainage surrounding the tumor. The compressed cortical veins are indicated by black arrows. **(F)** MFI delineating the course of the cortical veins, which were identified and preserved during surgery.

*Case 3*: A 48-year-old female presented with preoperative left-hand weakness. DSA demonstrated impaired cortical venous drainage caused by tumor-related compression of the central cortical veins. MFI further delineated the course of the involved cortical veins and their relationship with the tumor surface. During surgery, these veins were identified and carefully preserved based on the preoperative MFI. The patient’s limb weakness improved postoperatively (Figure 4).

**Figure 5:**
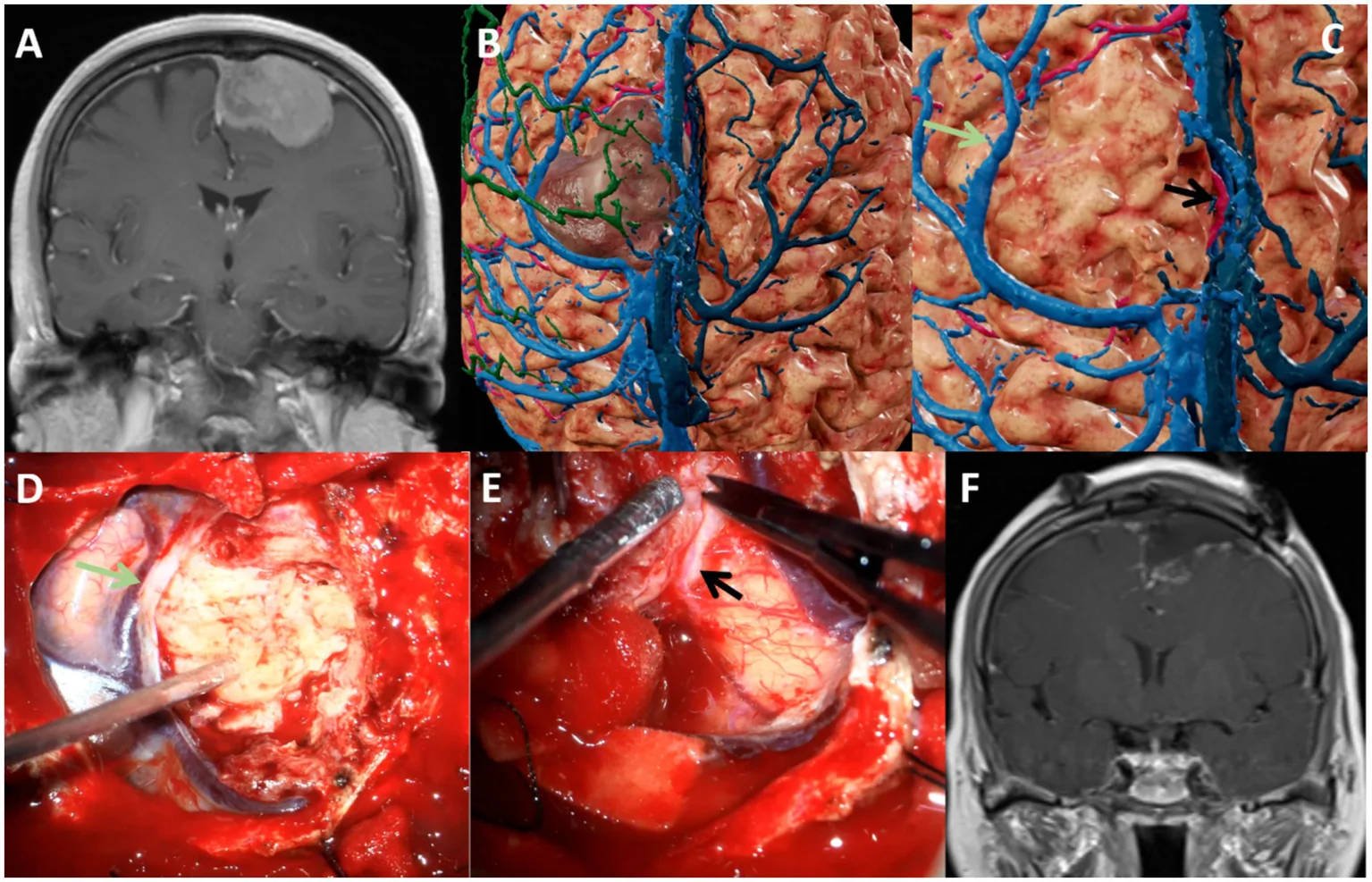
Parasagittal meningioma involving the superior sagittal sinus and adjacent cortical veins. **(A)** Preoperative contrast-enhanced MRI showing a left parasagittal meningioma. **(B–E)** Preoperative MFI and intraoperative comparison showing the middle meningeal artery, arteries, veins, tumor-associated cortical veins, and functional-zone arteries at the tumor base. The tumor-associated cortical vein is indicated by the green arrow, and the artery at the tumor base is indicated by the black arrow. **(F)** Postoperative contrast-enhanced MRI showing Simpson grade II resection.

*Case 4*: A 50-year-old female presented with right limb weakness and was diagnosed with a left parasagittal meningioma with superior sagittal sinus involvement. Preoperative MFI showed the relationship between the tumor, anterior cerebral artery, cortical veins, and superior sagittal sinus. These vascular relationships were visually validated after dural opening and were used to guide tumor dissection and vascular preservation. Simpson grade II resection was achieved (Figure 5).

**Figure 6:**
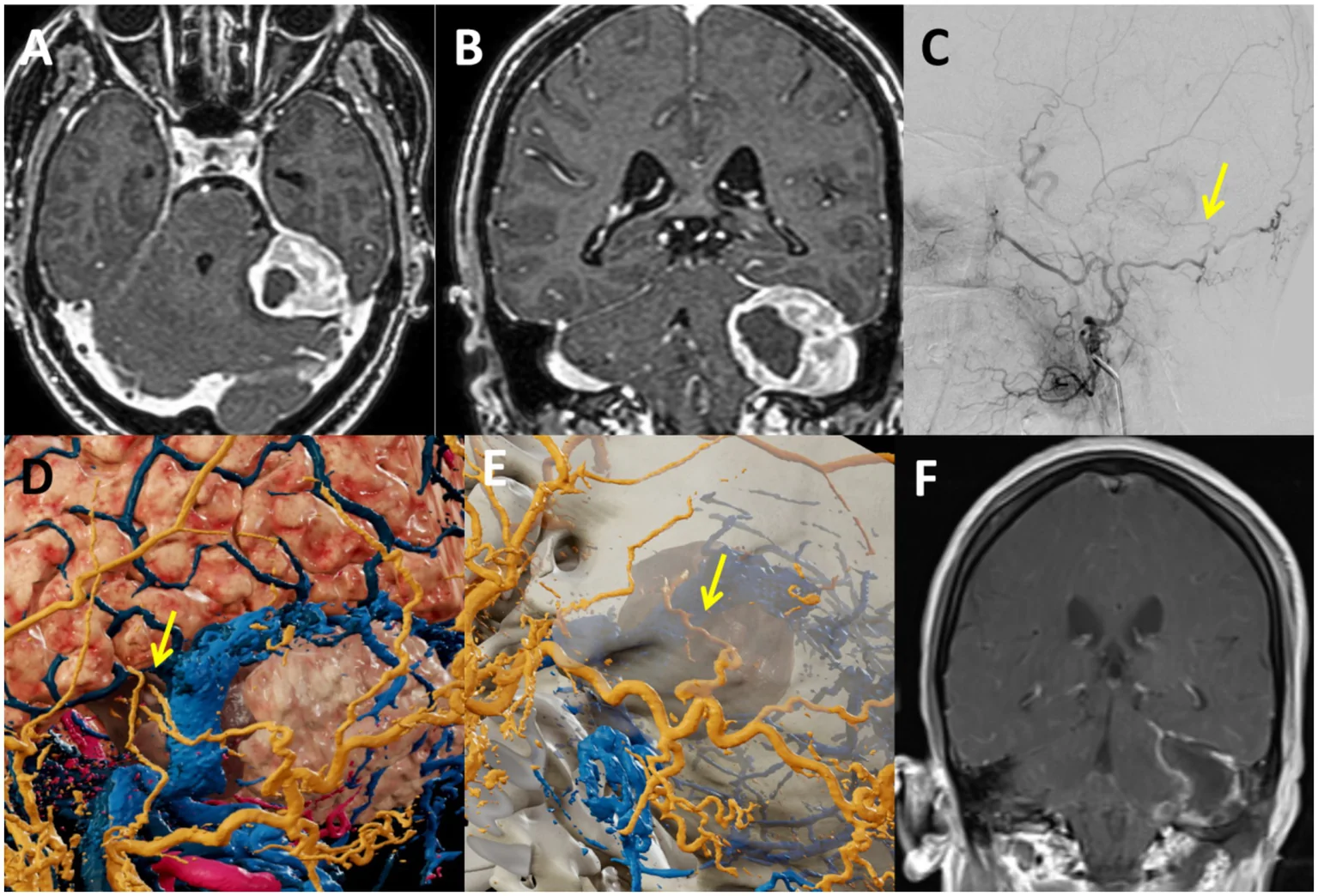
Tentorial meningioma supplied by a dural branch of the occipital artery. **(A,B)** Preoperative MRI showing a tentorial meningioma. **(C)** Preoperative DSA demonstrating tumor-feeding arteries originating from the dural branch of the occipital artery. The feeding arteries are indicated by yellow arrows. **(D,E)** MFI confirming that the feeding arteries traversed the petrous bone to reach the tumor base in the tentorial region, supporting selection of the presigmoid approach. **(F)** Postoperative MRI confirming Simpson grade I resection.

*Case 5*: A 50-year-old female presented with dizziness and was diagnosed with a tentorial meningioma. Preoperative DSA and MFI demonstrated that the tumor was supplied by a dural branch of the occipital artery, which traversed the petrous bone to reach the tentorial tumor base. The fused model clearly showed the tumor base and feeding artery trajectory, supporting the selection of a presigmoid approach. Postoperative MRI confirmed Simpson grade I resection (Figure 6).

## Discussion

Multimodal imaging fusion has become an important adjunct in neurosurgical planning by transforming separate two-dimensional datasets into patient-specific three-dimensional anatomical models (5, 6, 8–10). In the present series, 3DRA-based MFI was technically feasible in all 72 patients and integrated tumor morphology, arterial supply, venous anatomy, venous sinuses, and skull base structures. The observed Simpson grade I-II resection rate and complication rate describe the surgical results of this selected cohort; the uncontrolled retrospective design does not permit attribution of these outcomes to the imaging technique.

The potential incremental value of the present workflow lies in combining arterial- and venous-phase 3DRA with MRI and CT within a common three-dimensional space. MRI remains the principal modality for defining tumor extent, edema, and neural anatomy, whereas CT delineates calcification, hyperostosis, skull base structures, and bone corridors. CTA and MRA provide noninvasive vascular information and are sufficient for many uncomplicated tumors. Conventional DSA provides high spatial and temporal vascular resolution but remains projection-based. In selected complex lesions, 3DRA-based fusion may facilitate simultaneous assessment of small feeding arteries, pial supply, cortical veins, venous sinus patency, collateral venous pathways, tumor, and overlapping bone. However, because no direct comparison with CTA, MRA, conventional DSA, or another fusion workflow was performed, superiority over these approaches cannot be inferred.

3DRA-based MFI should not be routinely applied to all patients with intracranial meningiomas. Selective use may be considered when detailed arterial or venous information is expected to influence preoperative assessment or surgical strategy, including tumors involving a major dural venous sinus, critical cortical draining veins, or deep venous structures; lesions with suspected pial arterial supply or major arterial encasement; functionally eloquent-region meningiomas; skull base, tentorial, torcular, parasagittal, or recurrent tumors; hypervascular lesions with complex feeding arteries; and cases being considered for preoperative embolization. In these selected situations, the practical value of the fused model extends beyond improved image visualization. By presenting the tumor, arterial supply, cortical and deep venous drainage, venous sinuses, skull base structures, and surrounding neurovascular anatomy within a single patient-specific three-dimensional space, the model may provide the surgeon with a more intuitive understanding of the operative anatomy before surgery. This information may assist in identifying opportunities for early devascularization, anticipating pial arterial supply, judging whether further dissection across a vascularized tumor–brain interface is appropriate or whether a small residual should be accepted to preserve critical pial or cortical vessels, planning the preservation of cortical and deep venous drainage, determining the management of an involved venous sinus, and selecting or refining the operative corridor and extent of skull base bone removal. The model may also provide a common visual platform for multidisciplinary discussion, preoperative rehearsal, and education of less experienced surgeons. These represent clinically relevant anatomical, decision-support, and educational functions of the workflow. However, because the study lacked a control group and did not prospectively record management changes, vascular structures preserved, or injuries avoided, it cannot establish that the workflow reduces arterial or venous complications or improves clinical outcomes.

The expected anatomical benefit must be weighed against arterial puncture, iodinated contrast exposure, radiation, procedural time, specialized equipment and software, operator requirements, potential procedural complications, and additional cost. Although no DSA-related complication occurred in this cohort, 72 uneventful procedures are insufficient to establish procedural safety or justify universal use.

Associated vascular abnormalities, including nine aneurysms and one arterial occlusion, were concurrently identified during catheter angiography. Many such abnormalities can also be detected using CTA or MRA. In the present workflow, angiography provided additional characterization of the vascular anatomy and its relationship with the planned surgical corridor; however, this study was not designed to compare the diagnostic accuracy of DSA with noninvasive vascular imaging.

Reliable clinical modeling depends on standardized image acquisition, appropriate arterial and venous phase timing, accurate multimodal registration, segmentation review, and structured quality control. The standardized 3DRA protocol used in this study was intended to improve consistency in arterial and venous visualization. Before surgery, the fused models were cross-checked against the source MRI, CT, and DSA datasets, with attention to tumor boundaries, skull base landmarks, major arteries, cortical veins, and venous sinuses. Nevertheless, registration and segmentation accuracy were assessed visually rather than quantitatively. Future optimization should include predefined model-acceptance criteria, quantitative registration-error measurements, inter-observer assessment, automated or semi-automated vessel segmentation, and explicit representation of uncertainty.

The term “meningioma modeling” also encompasses biological and computational approaches. Khan et al. reviewed cell-based and animal models together with radiomics, artificial intelligence, and neural-network approaches used to investigate biological heterogeneity and treatment response (11). These approaches address a different but complementary level of care from the patient-specific anatomical MFI evaluated here. A clinically integrated pathway could use anatomical fusion models for operative and endovascular planning, while pathological, molecular, radiomic, and longitudinal data inform recurrence-risk assessment, adjuvant treatment, and surveillance. Such integration requires external validation and was beyond the scope of the present anatomical feasibility study.

Another feature of our workflow was continuity among angiographic acquisition, reconstruction, preoperative interpretation, and surgery. All angiographic acquisitions were performed by the same member of the surgical team, who also participated in reconstruction and the craniotomy procedures. This arrangement facilitated translation of vascular findings into the operative field and may have reduced communication gaps. At the same time, it may have introduced operator-dependent bias and limits conclusions regarding reproducibility in other teams or institutions.

In this cohort, meningeal arterial supply was identified in 54 patients, pial arterial supply in 14 patients, and venous sinus involvement in 24 patients. Identification of meningeal feeders may help anticipate the tumor base and possible sites for early devascularization. Recognition of pial supply may indicate a more complex tumor–brain interface and the need for careful preservation of cortical arteries. Venous-phase 3DRA allowed visualization of sinus patency, sinus invasion, cortical venous displacement, and collateral venous drainage. These relationships are particularly relevant for parasagittal, falcine, tentorial, torcular, and skull base meningiomas, in which decisions regarding sinus preservation, limited resection, or reconstruction may depend on the available venous pathways (12–17).

Abnormal vascular findings were detected in 10 patients, including 9 aneurysms and 1 arterial occlusion. These findings may influence perioperative risk assessment when surgery is planned near major arteries, skull base corridors, or venous sinuses. However, the present study did not systematically record whether these findings changed the operative or endovascular strategy.

The case illustrations demonstrate potential decision-support applications, including selection or confirmation of the operative corridor, planning of skull base drilling, identification of feeding arteries, and preservation of cortical or sinus drainage. These examples should not be interpreted as proof of improved outcomes. Changes in surgical or embolization planning were not prospectively recorded using predefined criteria, and estimated blood loss, operative duration, transfusion requirements, standardized functional outcomes, and cost-effectiveness were not systematically assessed. The resection and complication rates should therefore be interpreted as descriptive cohort outcomes rather than evidence that 3DRA-based MFI improves operative efficiency, extent of resection, neurological outcomes, or safety.

The potential role of 3DRA-based MFI in WHO grade 3 meningiomas requires separate consideration. Only three patients in this cohort had grade 3 tumors, precluding meaningful grade-specific analysis. In an international multicenter cohort, grade 3 meningiomas showed frequent recurrence and limited progression-free survival, and postoperative radiotherapy was associated with improved overall survival (18). These observations emphasize that advanced meningioma management extends beyond anatomical surgical planning. For aggressive or recurrent tumors, patient-specific mapping may assist repeat surgery, assessment of major-vessel or venous sinus relationships, definition of residual tumor adjacent to critical structures, and communication within a multidisciplinary team. However, MFI cannot determine histological grade, molecular alterations, recurrence risk, or treatment response and should complement, rather than replace, pathological and molecular assessment, radiotherapy planning, and longitudinal surveillance.

More broadly, multimodal image registration and fusion have been explored for neurosurgical planning across brain and skull base lesions (19, 20), while three-dimensional cerebral angiography has also been incorporated into multimodal surgical planning in other neurosurgical settings (21). The general fusion concept may also be applied to other intracranial tumors, including gliomas, but the reconstruction priorities would differ. Meningiomas are predominantly extra-axial lesions for which dural attachment, skull base anatomy, meningeal arterial supply, cortical veins, and venous sinuses are major determinants of strategy. Diffuse gliomas are intra-axial and may extend beyond contrast-enhancing margins. A glioma-oriented model would require greater emphasis on T2/FLAIR abnormalities, functional cortex, white-matter tracts, deep and perforating arteries, cortical and deep venous drainage, and infiltrative tumor regions. Diffusion tractography, functional MRI, perfusion imaging, and metabolic imaging may therefore be incorporated. Arterial- and venous-phase 3DRA may provide complementary information in selected deep-seated, insular, highly vascular, or vessel-adjacent gliomas, as previously described for insular glioma navigation (14), but catheter angiography would not be appropriate routinely.

Brain shift is a greater concern in intra-axial tumor surgery and is also an inherent limitation of any static preoperative model. After cerebrospinal fluid release, cortical opening, tumor decompression, or brain relaxation, the spatial relationship between the model and operative field may change. In glioma surgery, integration with intraoperative ultrasound, intraoperative MRI, updated navigation, fluorescence guidance, and functional mapping may therefore be required. In the present meningioma series, intraoperative validation was based on direct visual comparison rather than blinded quantitative image-to-field registration. Accordingly, 3DRA-based MFI should be regarded as an adjunct for preoperative planning and early anatomical orientation, not as a substitute for surgical judgment or real-time imaging.

This study has several limitations. It was retrospective, single-center, and uncontrolled, and the decision to perform 3DRA-based MFI was based on clinical judgment, introducing selection bias. The workflow was not directly compared with MRI alone, CTA, MRA, conventional DSA, or another fusion strategy. Changes in surgical approach, craniotomy design, embolization strategy, devascularization sequence, and venous preservation plan were not prospectively recorded. Estimated blood loss, operative duration, transfusion requirements, standardized functional outcomes, radiation dose, procedural cost, and cost-effectiveness were not systematically assessed. Registration and segmentation accuracy were evaluated visually rather than quantitatively or blindly. All acquisitions, reconstructions, and intraoperative validations were closely integrated within one surgical team, limiting assessment of reproducibility. The sample size was modest, only three tumors were WHO grade 3, and subgroup analyses were underpowered. Finally, the workflow requires catheter angiography, specialized software, standardized acquisition, and experienced operators, which may limit generalizability.

Despite these limitations, the study demonstrates the feasibility of integrating MRI, CT, arterial-phase 3DRA, and venous-phase 3DRA for patient-specific neurovascular and skull base assessment. The technique may be useful as a selective adjunct when complex arterial, venous, sinus, or osseous relationships are expected to affect surgical or embolization planning. Prospective controlled studies should compare this approach with noninvasive imaging strategies and include predefined management changes, operative time, blood loss, extent of resection, neurological and functional outcomes, radiation exposure, procedural complications, and cost-effectiveness.

Future studies should adopt a prospective comparative design with an appropriate control group undergoing standard preoperative imaging assessment. To directly evaluate the incremental effect on clinical decision-making, the intended surgical approach, craniotomy or skull base bone-removal strategy, vascular management plan, and indication or strategy for preoperative embolization should be documented prospectively before and after review of the 3DRA-based fusion images. This would allow management changes attributable to the additional imaging information to be quantified. Predefined clinical endpoints should include estimated blood loss, operative duration, transfusion requirements, extent of resection, perioperative neurological complications, standardized functional outcomes, DSA-related complications, radiation and contrast burden, and cost-effectiveness. Stratification according to tumor location, major arterial or venous involvement, vascular complexity, suspected pial arterial supply, and consideration of preoperative embolization may further identify the patient subgroups in whom the incremental value of 3DRA-based MFI is most likely to justify its invasiveness and resource requirements.

## Conclusion

Three-dimensional rotational angiography–based multimodal fusion imaging was technically feasible for patient-specific neurovascular and skull base assessment in this selected cohort of patients with anatomically complex intracranial meningiomas. By integrating arterial- and venous-phase 3DRA with MRI and CT, the workflow provided an intuitive three-dimensional representation of tumor-related arterial supply, cortical and deep venous drainage, venous sinuses, major vessels, and osseous anatomy. Its potential role may be most relevant in selected cases in which these anatomical relationships are expected to support surgical approach selection, arterial devascularization, venous preservation, sinus management, or embolization planning.

## Data Availability

The original contributions presented in the study are included in the article/supplementary material, further inquiries can be directed to the corresponding author.

## References

[1] Aldape K Brindle KM Chesler L Chopra R Gajjar A Gilbert MR . Challenges to curing primary brain tumours. Nat Rev Clin Oncol. (2019) 16:509–20. doi: 10.1038/s41571-019-0177-5, 30733593 PMC6650350

[2] Vadhavekar NH Sabzvari T Laguardia S Sheik T Prakash V Gupta A . Advancements in imaging and neurosurgical techniques for brain tumor resection: a comprehensive review. Cureus. (2024) 16:e72745. doi: 10.7759/cureus.72745, 39618625 PMC11607568

[3] Liao CC Wu KH Chen G. Application of preoperative multimodal image fusion technique in microvascular decompression surgery via suboccipital retrosigmoid approach. World Neurosurg. (2023) 173:e37–47. doi: 10.1016/j.wneu.2023.01.088, 36716853

[4] Aydin SO Barut O Yilmaz MO Sahin B Akyoldas G Akgun MY . Use of 3-dimensional modeling and augmented/virtual reality applications in microsurgical neuroanatomy training. Oper Neurosurg. (2023) 24:318–23. doi: 10.1227/ons.0000000000000524, 36701556

[5] Hirano T Ichikawa K Wanibuchi M Mikami T Suzuki J Nagahama H . Accuracy of computed tomography: magnetic resonance imaging image fusion using a phantom for skull base surgery. J Neurosurg Sci. (2022) 66:9–16. doi: 10.23736/S0390-5616.19.04621-6, 30808859

[6] Oishi M Fukuda M Hiraishi T Yajima N Sato Y Fujii Y. Interactive virtual simulation using a 3D computer graphics model for microvascular decompression surgery. J Neurosurg. (2012) 117:555–65. doi: 10.3171/2012.5.JNS112334, 22746377

[7] Zhou L Wang W Wei H Song P Li Z Cheng L . Clinical application of 3D slicer combined with Sina/MosoCam multimodal system in preoperative planning of brain lesions surgery. Sci Rep. (2022) 12:19258. doi: 10.1038/s41598-022-22549-7, 36357434 PMC9649692

[8] Barillot C Lemoine D Le Briquer L Lachmann F Gibaud B. Data fusion in medical imaging: merging multimodal and multipatient images, identification of structures and 3D display aspects. Eur J Radiol. (1993) 17:22–7. doi: 10.1016/0720-048X(93)90024-H, 8348908

[9] Gandhe AJ Hill DL Studholme C Hawkes DJ Ruff CF Cox TC . Combined and three-dimensional rendered multimodal data for planning cranial base surgery: a prospective evaluation. Neurosurgery. (1994) 35:463–70. doi: 10.1227/00006123-199409000-00015 7800138

[10] Jian ZH Li JY Wu KH Li Y Li SX Chen HD . Surgical effects of resecting skull base tumors using pre-operative multimodal image fusion technology: a retrospective study. Front Neurol. (2022) 13:895638. doi: 10.3389/fneur.2022.895638, 35645981 PMC9133916

[11] Khan M Hanna C Findlay M Lucke-Wold B Karsy M Jensen RL. Modeling meningiomas: optimizing treatment approach. Neurosurg Clin N Am. (2023) 34:479–92. doi: 10.1016/j.nec.2023.02.014, 37210136

[12] Oishi M Fukuda M Yajima N Yoshida K Takahashi M Hiraishi T . Interactive presurgical simulation applying advanced 3D imaging and modeling techniques for skull base and deep tumors. J Neurosurg. (2013) 119:94–105. doi: 10.3171/2013.3.JNS121109, 23581591

[13] Sato M Tateishi K Murata H Kin T Suenaga J Takase H . Three-dimensional multimodality fusion imaging as an educational and planning tool for deep-seated meningiomas. Br J Neurosurg. (2018) 32:509–15. doi: 10.1080/02688697.2018.1485877, 29943649

[14] Dasenbrock HH See AP Smalley RJ Bi WL Dolati P Frerichs KU . Frameless stereotactic navigation during insular glioma resection using fusion of three-dimensional rotational angiography and magnetic resonance imaging. World Neurosurg. (2019) 126:322–30. doi: 10.1016/j.wneu.2019.03.096, 30898738

[15] Conti A Pontoriero A Faragò G Midili F Siragusa C Granata F . Integration of three-dimensional rotational angiography in radiosurgical treatment planning of cerebral arteriovenous malformations. Int J Radiat Oncol Biol Phys. (2011) 81:e29–37. doi: 10.1016/j.ijrobp.2010.12.024, 21345616

[16] Yoshino M Nakatomi H Kin T Saito T Shono N Nomura S . Usefulness of high-resolution 3D multifusion medical imaging for preoperative planning in patients with posterior fossa hemangioblastoma: technical note. J Neurosurg. (2017) 127:139–47. doi: 10.3171/2016.5.JNS152646, 27564468

[17] Srinivasan VM Schafer S Ghali MG Arthur A Duckworth EA. Cone-beam CT angiography (dyna CT) for intraoperative localization of cerebral arteriovenous malformations. J Neurointerv Surg. (2016) 8:69–74. doi: 10.1136/neurintsurg-2014-011422, 25480885

[18] Tosefsky K Rebchuk AD Wang JZ Ellenbogen Y Drexler R Ricklefs FL . Grade 3 meningioma survival and recurrence outcomes in an international multicenter cohort. J Neurosurg. (2024) 140:393–403. doi: 10.3171/2023.6.JNS23465, 37877968

[19] Nandish S Prabhu G Rajagopal KV. Multiresolution image registration for multimodal brain images and fusion for better neurosurgical planning. Biom J. (2017) 40:329–38. doi: 10.1016/j.bj.2017.09.002, 29433836 PMC6138619

[20] Jian ZH Chen P Li Y Liao CC Yi XF Zhan RG . Surgical management of complex skull base tumor using preoperative multimodal image fusion technology. J Craniofac Surg. (2024) 35:853–9. doi: 10.1097/SCS.0000000000010073, 38534161 PMC11045550

[21] Cardinale F Pero G Quilici L Piano M Colombo P Moscato A . Cerebral angiography for multimodal surgical planning in epilepsy surgery: description of a new three-dimensional technique and literature review. World Neurosurg. (2015) 84:358–67. doi: 10.1016/j.wneu.2015.03.028, 25819527

